# Bioinspired Sensor Systems

**DOI:** 10.3390/s111110180

**Published:** 2011-10-26

**Authors:** Manel del Valle

**Affiliations:** Sensors and Biosensors Group, Department of Chemistry, Universitat Autònoma de Barcelona, 08193 Bellaterra, Spain; E-Mail: manel.delvalle@uab.es; Tel.: +34-93-581-1017; Fax: +34-93-581-2477

**Keywords:** artificial vision, electronic nose, electronic tongue, swarm sensor network, body sensor network

## Abstract

This editorial summarizes and classifies the contributions presented by different authors to the special issue of the journal *Sensors* dedicated to Bioinspired Sensor Systems. From the coupling of sensor arrays or networks, plus computer processing abilities, new applications to mimic or to complement human senses are arising in the context of ambient intelligence. Principles used, and illustrative study cases have been presented permitting readers to grasp the current status of the field.

## 1. Introduction

Nature has developed, through thousands of years of evolution, sensing organs and strategies that stand out in versatility, performance, sensitivity or tolerance to saturation. Curiously, the strategies followed by Nature are quite peculiar. First, receptors in sensor buds are not of high specificity, but of broad response; second, animal senses use bunches of slightly different receptors to allow by combinatorial principles to discriminate between thousands of targets. With these concepts, a generic sensorial detection event is formed by a large number of individual, imperfect receptor responses that are next processed by an associated nervous system; this is common from the simple insect to the human being; and it is the processing of this multivariate information that provides the high level, specialized response [1]. From these principles, the electronic nose, formed by slightly different sensor arrays to perform analysis in the gas phase and closely related to artificial olfaction stands out, or the electronic tongues, using arrays of sensors, that can be of different nature and are applied with liquid samples; these can also be devised to mimic animal taste. The sensor array principle may be extended to the use of biosensors, in search of improving their intrinsically superior selectivity, where they can offer interesting options for medical diagnostic or security threats. But the same principle of using sets of sensors is being adapted to many other objectives: for example, a skin of pressure sensors can be employed in array mode to detect structural stress in vehicles or buildings. In connection to this, biology offers very interesting operating principles to locate a source from an odor plume, or to track a fish prey from some trail scent, of uppermost interest in the case of locating victims of catastrophes or explosives. Also related are the sensor networking principles, aimed at detecting the entrance of a chemical (or an intruder), that can be devised from common principles used by swarms: to operate a number of sensing devices deployed along a region, and establish simple communication between them to locate the spot. The special issue of the journal *Sensors* will cover these different aspects of biologically inspired sensing and networking, which surely will be of interest to readers seeking to grasp the lastest trends in the field.

## 2. Developments in Bionspired Sensor Systems

The different contributions related to bioinspired techniques for (bio)sensors have been classified following the main human senses into tactile, vision applications, olfaction, and taste developments plus specific biosensing strategies and bioinspired networked systems, commented at the end.

### 2.1. Tactile Applications

Tactile sensors are basically arrays of force sensors that are intended to emulate the skin in applications such as assistive robotics. A first paper dealing with the topic describes a programmable system devised with the use of piezoresistive tactile sensors [2]. Very much in connection with tactile sensing is the recognition of fingerprints. The work of Xie *et al*. describes an effective fingerprint quality estimation system that can be adapted for different types of capture devices, like optical, capacitive and thermal sensors, and which is designed by modifying and combining a set of features including orientation certainty, local orientation quality and consistency [3]. The proposed system extracts basic features, and generates next level features which are applicable for various types of capture sensors. The system then uses the Support Vector Machine (SVM) classifier to determine whether or not an image should be accepted as input to the recognition system. A related work [4] investigates the influence of fingerprints and their curvature in tactile sensing performance by comparative analysis of different design parameters in a biomimetic artificial fingertip, having straight or curved fingerprints. Recognition ability was studied varying normal contact force and tangential sliding velocity, as a function of fingertip rotation along the indentation axis.

### 2.2. Vision Applications

A first interesting research reports a bioinspired electronic white cane for blind people using the whiskers principle for short-range navigation and exploration [5]. The raw data were generated from a low-size terrestrial LIDAR and a tri-axial accelerometer, which were then converted into tactile information using several electromagnetic devices configured as a tactile belt for user feedback. Also connected to this is the motion estimation, a low-level vision task especially relevant due to the wide range of applications [6]. The work describes a bioinspired motion estimation algorithm employing optical flow and orthogonal image variant moments, capable of producing a mid-level vision abstraction layer.

### 2.3. Olfaction Developments

The electronic nose (e-nose) crudely mimics the mammalian nose in furnishing a sensor array that non-selectively interact with odor molecules to produce some sort of signal that is then sent to a computer that uses multivariate statistics to determine patterns in the data. In a pattern recognition implementation, qualitative identification of a sample from its headspace volatiles can be accomplished. A first review paper in this section [7] describes the families of sensors used in e-noses, including organic polymers, metal oxides, quartz crystal microbalance and even gas-chromatography (GC) or combined with mass spectroscopy (MS) that can be used in a non-selective manner using chemical mass or patterns from a short GC column as an e-nose or “Z” nose. The review focuses on applications of the e-nose technology for edible products and pharmaceutical uses. A continuing manuscript by Mamat *et al*. reports an e-nose prototype for reliable measurement and correct classification of beverages [8], which was developed using commercially available metal oxide gas sensors and a temperature sensor. Performance of the developed e-nose was tested on odors emanating from different beverages such as blackcurrant juice, mango juice and orange juice, respectively. The e-nose prototype was also used successfully to differentiate beverages such as milks treated with different heat treatments (ultra high temperature, pasteurization) and fresh and spoiled milks. The discriminative ability of the e-nose was evaluated using Principal Component Analysis (PCA) and a Multi Layer Perceptron Neural Network. A more elaborate and specific work is that of Campagnoli *et al*. [9] that has studied detection of mycotoxin contamination of cereal grains employing a commercial e-nose equipped with metal-oxide-semiconductor (MOS) sensors and a trap with thermal desorption technique. The e-nose was able to detect durum wheat whole-grain samples naturally contaminated with deoxynivalenol at the concentration level recommended by the European legislation (1.750 μg/kg), employing PCA processing and a classifier based on classification and regression trees (CART).

### 2.4. Taste Developments

Similar to the e-nose is the electronic tongue (e-tongue) that, according to IUPAC [10], is defined as “a multisensor system, which consists of a number of low-selective sensors and uses advanced mathematical procedures for signal processing based on Pattern Recognition and/or Multivariate data analysis—Artificial Neural Networks (ANNs), Principal Component Analysis (PCA), *etc*”. This research area, which is receiving increased attention in recent years [11], presents ample variety of sensors principles or processing variants, but also in the type of applications devised, where it goes from the qualitative identification of beverages or food types, to the approximation of automatic taste perception [12].

A first paper on this subject describes the use of an array of potentiometric sensors and an ANN response model to determine perchlorate and sulfide ion mixtures in polluted waters [13]. The study case illustrates the potential use of e-tongues in the quantification of mixtures when interfering effects need to be counterbalanced: relative errors in determination of individual ions can be decreased typically from 25% to less than 5%, if compared to the use of a single proposed ion-selective electrode. Also with the use of potentiometric sensors is the review paper [14] dedicated to overview various examples of the design, performance, and applications of different e-tongue systems designed by the Department of Microbioanalytics at the Warsaw University of Technology (WUT).

Although with some added treatment complexity, e-tongues can also be devised using sensors of the voltammetric type. The next work [15] describes the sensing properties of carbon paste electrodes (CPEs) prepared from three different types of carbonaceous materials: graphite, carbon microspheres and carbon nanotubes, and their electrochemical responses to different antioxidants. When these electrodes are used as an array, a PCA treatment of the generated data permitted to discriminate among different antioxidants according to their chemical structure and reactivity.

With a higher degree of complexity, there are also e-tongue systems combining sensors from different types to provide better discrimination ability. One sample of these hybrid e-tongues [16] is the one formed by five types of microfabricated sensors: ISFET, conductivity, redox potential, voltammetric and optochemical, applied in this case [17] to characterize and classify white wines according to their grape variety and geographical origin. PCA and Soft Independent Modeling Class Analogy (SIMCA) were used for qualitative identification, while Partial Least Squares (PLS) was the tool used for quantitative estimation.

Lastly, in the greater level of complexity, there is the possibility of coupling gas sensors and liquid sensors, that is, the e-nose plus the e-tongue, in a sensor fusion approach to better tackle the most demanding situations. This is the case of the presented work aimed to the classification of honeys of different floral origin and the detection of its adulteration [18]. It was thanks to the multi-modality sensor fusion that the effective discrimination of samples was possible, in a way mimicking how humans perceive tastes, flavours and aromas.

### 2.5. Biosensor Applications

An inhibition bioelectronic tongue to determine pesticide mixtures is presented [19], in this case representing an interesting use of amperometric enzyme biosensors as an e-tongue. The inhibition method was based in the automated injection of the insecticide when the enzyme activity has reached a steady state current with an initial level of substrate. Operation of the sensors was accomplished with a Multi-commutated Flow Analysis system (MCFA), permitting the discrimination of the insecticides chlorpyrifos oxon, chlorfenvinfos and azinphos methyl-oxon.

One interesting application of biosensors in developing biological sensors is the estimation of risk of living organisms after exposure to toxic agents or conditions. In this case, the uses of biosensor principles for monitoring the harmful impact of solar UV radiation have been reviewed [20]. The different variants that can be employed to measure the biological effects of the UV sunlight are described, as those based on DNA damage, effect to bacteria or even mammalian cells.

A further bioinspired biosensing is the research work dealing with gas biosensing based on a cytochrome c biosensor [21]. Emphasis is put on the analysis of the sensing process and a mathematical model to make predictions about the biosensor response. Applicability of the work developed was demonstrated for the particular example of detection of methanethiol.

Finally a surface plasmon resonance (SPR) biosensor with immobilized specific probe is used to study the aggregation process of amyloid-β peptide (Aβ40) in order to find potential anti-neurodegenerative drugs [22]. Among the substances initially screened are the alkaloids recoline hydrobromide, pseudopelletierine hydrochloride, trigonelline hydrochloride and α-lobeline hydrochloride.

### 2.6. Networked Systems

Biology has often been used as a source of inspiration in computer science and engineering. Bioinspired principles have found their way into network node design and research due to the appealing analogies between biological systems and large networks of small sensors. A first paper devoted to networked systems [23] provides an overview of bioinspired principles and methods such as swarm intelligence, natural time synchronization, artificial immune system and intercellular information exchange applicable for sensor network design.

A closely related research paper describes a swarming mobile sensor network comprised of a swarm of wirelessly connected mobile robots equipped with various sensors [24]. Such a network can be applied in an uncertain environment for services such as cooperative navigation and exploration, object identification and information gathering. The paper describes a method for link optimization based on combination of the artificial potential force guaranteeing connectivities of the mobile sensor nodes and the max-flow min-cut theorem of graph theory ensuring optimization of the network link capacity.

A specific field of application for the sensor network technologies is the healthcare sector. In it, a in the existing evolution for continuous monitoring of patients is recent trend is the use of wearable and implantable body area network systems [25]. Body sensor networks are foreseen to minimize the need for caregivers and to help the chronically ill or the elderly people living an independent life, besides providing people with proper care and quality of life. Continuing with the latter, a wireless body sensor network, successfully tested on a FPGA board [26], is developed with low power consumption and capable of monitoring and transmitting various biomedical signals.

In conclusion, this special issue gathers representative indicators of the research being done in biologically inspired sensing. The trends sketched in this collection of papers clearly indicates progressive use of computer processing with use of biological models and strategies in all stages of (bio)sensing and/or their networking, a trend that for sure will continue its activity and show further developments in the next future.

## Figures and Tables

**Figure 1.**
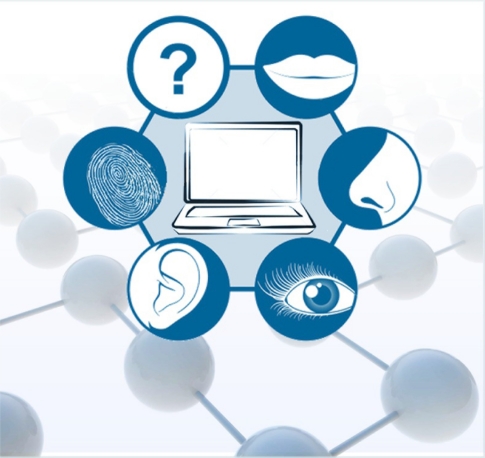
Sensing principles inspired in animal senses and/or networking are revolutionizing performance and scope of application of sensor systems.
